# The Relationship Between Ankle Proprioception and Functional Mobility in People With Parkinson's Disease: A Cross-Sectional Study

**DOI:** 10.3389/fneur.2020.603814

**Published:** 2021-01-15

**Authors:** Yejun Wang, Jeremy Witchalls, Elisabeth Preston, Zhen Wang, Jie Zhuang, Gordon Waddington, Roger Adams, Jia Han

**Affiliations:** ^1^Department of Sport Rehabilitation, Shanghai University of Sport, Shanghai, China; ^2^Research Institute for Sport and Exercise, University of Canberra, Canberra, ACT, Australia; ^3^Faculty of Health, University of Canberra, Canberra, ACT, Australia; ^4^College of Chinese Wushu, Shanghai University of Sport, Shanghai, China

**Keywords:** neurodegenerative diseases, walking speed, ankle joint, gait, time and motion studies

## Abstract

Previous research has found ankle proprioception to be impaired in people with Parkinson's disease (PD). However, the relationship between ankle proprioception and functional mobility in people with PD has not been fully investigated. The purpose of this study was to examine whether ankle proprioception is related to the functional mobility of people with PD. Forty-two participants with mild to moderate PD volunteered. Ankle proprioceptive acuity was measured in standing, by using active movement extent discrimination assessment (AMEDA). Functional mobility measures included the timed-up-and-go test (TUG), 30 s sit-to-stand test (30s-STS) and 10-meter walking test (10MWT). Step length and step cadence were recorded during the 10MWT. No significant correlation was found between ankle proprioceptive discrimination scores and any mobility performance measure in people with PD (−0.20<r<0.04, all *p* > 0.05). However, ankle proprioception scores were significantly correlated with step length (*r* = 0.38, *p* < 0.05) and step cadence (*r* = −0.30, *p* < 0.05), and were significantly and negatively correlated with the stage of modified Hoehn and Yahr (rho = −0.53, *p* < 0.01). The lack of relationship between ankle proprioceptive acuity and functional mobility in PD suggests that people with PD may be more limited by reduced sensorimotor integration or may rely more on other sensory input, rather than ankle proprioception, to achieve functional mobility, a finding consistent with sensory reweighting theory. In addition, poorer ankle proprioceptive acuity was associated with decreased step length and increased step cadence, suggesting that the shuffling gait observed in PD may be related to impaired ankle proprioception, which has important clinical implications for gait retraining in people with PD. Given that ankle proprioception was significantly and negatively correlated with the stage of modified Hoehn and Yahr, it may warrant being used as an objective biomarker to monitor the progression of PD.

## Introduction

Parkinson's disease (PD) is the second most common neurodegenerative disease and may impair patients' functional mobility (1–3). Functional mobility has been defined as the physical ability to move safely and independently in different environments, in order to complete daily life activities or specific tasks (4–6). Although there are various physical and psychological factors that may affect the mobility of people with PD (5), decreased functional mobility in people with PD has been attributed to abnormal neural activity in the basal ganglia that may significantly decrease the precision of motor control, in two ways (7, 8). First, because one-third of the neurons in the basal have proprioceptive fields basal ganglia dysfunction may reduce the quality of afferent proprioceptive information, which may result in poor functional mobility in people with PD. On the other hand, basal ganglia also play an important role in sensorimotor integration, which is vital for movement planning and execution (8, 9). Thus, impaired integration of available sensorimotor information could also negatively affect functional mobility of people with PD. To date, however, the neural mechanisms underlying impaired functional mobility in people with PD are still unclear.

Motor impairments such as hypokinesia, bradykinesia and akinesia have already been recognized in people with PD (2, 10), however, the role of sensory inputs for motor performance in this group is still unclear. Proprioception, one of important source of sensory information, is essential for motor control in healthy adults (11–13). Our previous research has shown that, compared to other lower limb joints, proprioception at the ankle contributes more significantly to sports performance in athletes (14–16), and to functional mobility in community older adults (17–20). With respect to people with PD, our previous study showed that, compared to a healthy control group, ankle proprioception was poorer in the PD group, and there was a moderate correlation between impaired ankle proprioception and the symptoms measured by Parkinson's Disease Questionnaire (PDQ-39) (21). Whether impaired ankle proprioception is associated with poor functional mobility in people with PD is currently undetermined, and relevant research could advance understanding of the neural mechanisms underlying impaired functional mobility in people with PD and inform effective interventions for functional mobility in people with PD.

One hypothesis is that impaired ankle proprioception would be significantly correlated with low functional mobility in people with PD (8), suggesting that proprioceptive information may be crucial for functional mobility in PD. However, according to sensory reweighting theory (12, 22, 23), diminished ankle proprioception may be not significantly correlated with functional mobility in people with PD, because the brain may be able to use other sensory inputs, such as visual and vestibular input to achieve effective functional mobility.

In order to test these hypotheses, we employed an ecologically valid, weight-bearing ankle proprioception test– the Active Movement Extent Discrimination Assessment (AMEDA), and a battery of functional mobility tests, including the timed-up-and-go test (TUG), 30 s sit-to-stand test (30s-STS) test and 10-meter walking test (10 MWT) to explore the relationship between ankle proprioception and functional mobility in people with PD. We hypothesized that ankle proprioception would be significantly correlated with functional mobility measurements and severity of the disease.

## Materials and Methods

### Participants and Design

The study involved 42 people with PD who were diagnosed by a neurological specialist according to the definition of the United Kingdom Disease Society (5). The study was approved by the Ethics committee at the Shanghai University of Sport (Approval number: 2016035). All participants provided signed informed consent before data collection. Disease severity was assessed by the stage of the modified Hoehn and Yahr (H-Y) (24) and the New Freezing of Gait Questionnaire (NFOG-Q) (25). Cognitive function was measured by the mini-mental state examination (MMSE). The inclusion criteria were: (1) walk 20 m independently without a walking aid; (2) maintain standing balance without support for at least 1 min; (3) have a MMSE score >24; (4) have had no lower limb injuries or operations in the last 6 months; (5) have no contraindications to performing the assessment tasks, such as severe coronary heart disease; (6) have no other neurological conditions or any vestibular or visual impairment; and (7) have no history of deep brain stimulation treatment.

### Assessment

Participants completed the following tests in a randomized order: ankle proprioception testing (AMEDA), functional mobility tests including the timed-up-and-go test (TUG), 30 s sit-to-stand test (30s-STS) test and 10-meter walking test (10 MWT). The primary outcome measures are ankle proprioception and 10 MWT. Secondary outcome measures are TUG and 30s-STS. All tests were conducted during participants' optimally medicated phase (21). Participants were allowed a rest between tests, and the total time to complete all tests was <30 min.

### Ankle Proprioception

**Figure 1:**
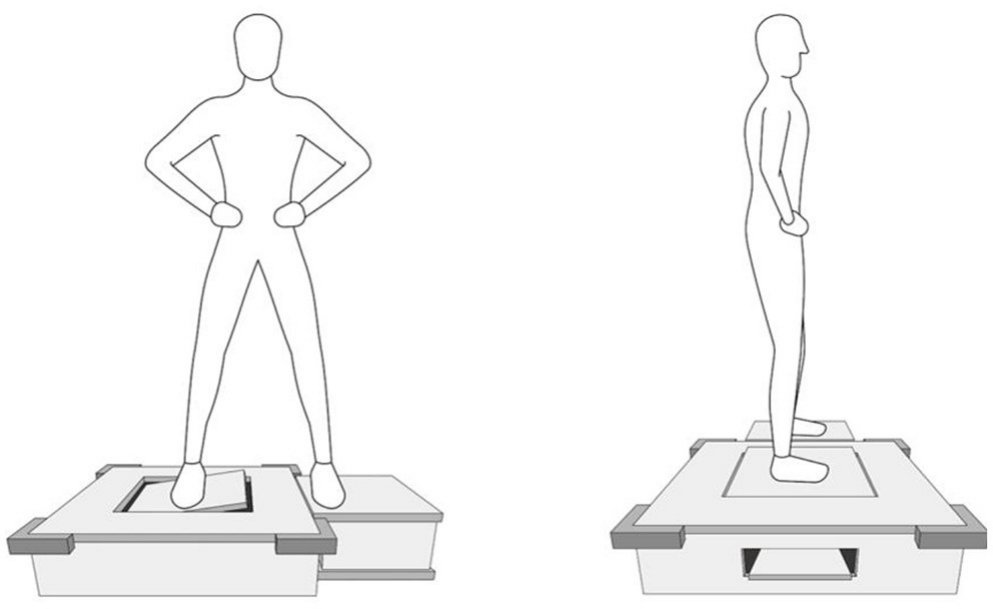
The Active Movement Extent Discrimination Assessment (AMEDA) for ankle proprioception in standing.

The ankle AMEDA system was used to test the participant's proprioception at the ankle joint in functional standing (13) (Figure 1). According to Han et al. proprioception is “an individual's ability to integrate the sensory signals from mechanical receptors to thereby determine body segment positions and movements in space.” Therefore, the ankle AMEDA is to assess the quality of this proprioception (13). The ankle AMEDA has shown good to excellent reliability for testing ankle proprioception in both older and young adults (16, 20). The AMEDA apparatus was used to generate a set of four end positions reached by active inversion movement. The four predetermined displacements of ankle inversion from smallest to largest were: 10, 12, 14, and 16°. A position number was assigned to each displacement in order (position 1=10°, position 2=12°, position 3=14° and position 4=16°) so that participants were able to use the assigned position numbers when making their responses during the test. During testing, the participant's focus was directed at the angle of their foot and ankle, but the AMEDA does permit some movement of other lower limb joints, and allows utilization of inputs from other sensory modalities such as vision and vestibular system. In so doing, the AMEDA gains ecological validity through being compatible with weight-bearing function in real-life activities (13). Before the beginning of the test, each participant underwent a standardized familiarization process, with exposure to positions one to four consecutively, repeated three times. During the test, each ankle inversion position was presented 10 times in random order. The response to a presented position was recorded, and from the response data, a receiver operating characteristic (ROC) curve was generated for each adjacent position pairing. The mean area under the ROC curve (AUC) was calculated to represent the participant's ability to discriminate between the four ankle inversion movement extents. The AUC proprioceptive discrimination sensitivity scores can range from 0.5 to 1.0, where 0.5 represents a chance level of responding and 1.0 implies that the participant has excellent ankle proprioception ability so as to perfectly discriminate between the four ankle inversion movement extents (13).

### Functional Mobility

#### Timed-Up-and-Go Test (TUG)

For the TUG test (26), the participants started from a sitting position. The time that participants needed to rise from a chair, walk three meters at their comfortable speed, turn around, walk back, and sit down was recorded. Participants practiced before the test to ensure that participants understand the testing procedure. Each participant was required to repeat the test 3 times and the average value was their score.

#### 30 Seconds Sit-to-Stand Test (30s-STS)

For the 30s-STS (27), participants placed arms crossed onto the opposite shoulder, kept feet flat on the floor, and sat upright on the middle of a chair with a height of 46 cm. Within 30 s participants were encouraged to safely complete as many full standing and sitting cycles as they could. There was one familiarization practice before the test to ensure that they understood the testing procedure. The number of times the patient completed the task in 30 s was recorded. Given that the task was extremely physically-demanding for people with PD, their performance for one 30s-duration trial was recorded.

#### 10-Meter Walking Test (10MWT)

During the 10 MWT (28), participants were asked to walk at their comfortable speed on a 10-meter walkway. For the 10 m distance, two end lines and two buffer lines were shown by tape on the ground. The distance between two end lines was 14 meters and each buffer line was 2 meters from the end line. The time to walk the middle 10 meters (between the two buffer lines) was recorded using a stopwatch. Each participant was required to repeat the test 3 times and the average value was used. During each test, the steps taken in the middle 10 meters were recorded by an observer. Average step length (m) and step cadence (step/min) were calculated.

### Statistical Analysis

All data analyses were performed using IBM SPSS Statistics 22.0. For the demographic, ankle proprioception and functional mobility performance measures, descriptive statistics (mean ± SD) were calculated. Pearson and Spearman Correlations were carried out to examine the correlations between ankle proprioception and functional mobility test variables. Pearson's Correlation was used for continuous variables, including the AUC ankle proprioception score, TUG, the 10 MWT time, step length on the 10 MWT, and step cadence on the 10 MWT. Spearman's Correlation was calculated for the AUC ankle proprioception score with the stage of modified Hoehn and Yahr scores, 30s-STS and score from the NFOG-Q. To compare characteristics, severity of disease, ankle proprioception scores and all functional mobility performance scores between male and female participants, independent-samples *t*-tests were conducted. The variables involved in independent *t*-tests were examined to determine that they were acceptably normally distributed.

## Results

The demographic information, severity of disease, and results from the ankle proprioception and functional mobility tests are given in Table 1. Compared to their female counterparts, male participants were significantly taller, heavier and had higher scores on the New Freezing of Gait Questionnaire. There was no significant between-gender difference for any other measure. Correlation analysis showed that, in PD, ankle proprioception was not significantly related to any functional mobility measure or NFOG-Q (−0.20<r<0.04, *p* > 0.05). However, moderate correlations were observed between ankle proprioception and step length from the 10 MWT test (*r* = 0.38, *p* < 0.05), and between ankle proprioception and step cadence from the 10 MWT test (*r* = −0.30, *p* < 0.05). In addition, ankle proprioception had a significant correlation with the stage of modified Hoehn and Yahr (rho = −0.53, *p* < 0.01).

**Table 1:** Characteristics, Severity of disease, Ankle proprioception and Functional mobility performance in people with PD.

	**Total (*n* = 42)**	**Male (*n* = 21)**	**Female (*n* = 21)**	***p***
**Characteristics**				
Age, y, mean ± SD	62.6 ± 6.2	64.0 ± 6.9	61.1 ± 5.3	0.14
Weight, cm, mean ± SD	62.3 ± 11.3	67.7 ± 10.2	56.9 ± 9.9	<0.01^**^
Height, cm, mean ± SD	162.4 ± 7.1	167.3 ± 4.1	157.5 ± 6.0	<0.01^**^
**Severity of disease**				
Stage of Modified H-Y, n				
1	17	8	9	
1.5	7	5	2	
2	9	3	6	
2.5	3	2	1	
3	6	3	3	
4	0	0	0	
5	0	0	0	
Score of NFOG-Q, (out of 28)	7.33 ± 7.67	9.76 ± 8.29	4.90 ± 6.27	0.04^*^
**Ankle proprioception**				
AMEDA, AUC	0.64 ± 0.09	0.65 ± 0.07	0.63 ± 0.11	0.52
**Functional mobility**				
TUG, s	9.62 ± 2.25	10.30 ± 2.50	8.95 ± 1.80	0.28
30s-STS, n	12.81 ± 3.40	12.24 ± 3.25	13.38 ± 3.53	0.58
10MWT, s	8.66 ± 1.60	8.95 ± 1.71	8.37 ± 1.46	0.25
Step length, m	0.62 ± 0.08	0.61 ± 0.08	0.62 ± 0.09	0.88
Step cadence, step/min	116.45 ± 14.66	112.78 ± 11.58	120.12 ± 16.67	0.84

* *Significant difference at the p < 0.05 level*;

** *Significant difference at the p < 0.01 level*.

*NFOG-Q, New Freezing of Gait Questionnaire; H-Y, Hoehn and Yahr; AMEDA, Active movement extent discrimination assessment; ankle proprioception testing; TUG, Timed-up-and-go test; 30s-STS, 30 seconds Sit-to-Stand test; 10MWT, 10-meter walking test*.

## Discussion

Impairments in proprioception and functional mobility are often seen in patients with PD, and these impairments are not commonly improved by medical treatments (5, 29, 30). In this study, ankle proprioception using AMEDA, and multiple functional mobility measurements were made in a group of patients with mild to moderate PD. Although no significant correlation was found between ankle proprioceptive acuity scores and any mobility performance measure in people with PD, ankle proprioception was significantly correlated with step length and step cadence on the 10 MWT. Participants with higher proprioception scores took longer and slower steps during the 10 MWT. In addition, ankle proprioceptive scores were significantly correlated with the stage of disease, measured using the modified Hoehn and Yahr scale.

Previous animal and human studies have established the influence of abnormal basal ganglia activity on proprioception in PD (8) and the progressive impairment in proprioception observed has been regarded as a cause of motor abnormalities in PD (8, 31). In the present study, however, the lack of significant correlation findings between ankle proprioception and functional mobility performance scores suggests that the poor performance of functional mobility, including walking and sit-to-stand transfer, seen in PD may not be directly attributable to impaired ankle proprioception, and this was contrary to our hypothesis. Rather, our results were consistent with recent studies that suggest that the contribution from each sensory system to motor control is a dynamic process, and that the CNS weights more reliable sensory information to a greater extent than information from less reliable sources (22, 23, 32, 33). Thus, in testing here, people with PD may have relied more on other sensory information, such as that arising from the visual or vestibular systems, to compensate for reduced ankle proprioceptive input (32, 34). Indeed, results from several studies support this notion. Suarez et al. (35) found that visual cues could significantly affect standing stability in people with PD, while no significant difference was found in a control group during Limit of Stability and body center of pressure. Likewise, during a reaching movement task, people with PD depended more on their visual system and therefore failed at the task when they could not see their arms (36). Therefore, it is possible that because of impairment to ankle proprioception in people with PD, the CNS may rely more on other sensory inputs, such as from the visual or vestibular systems, and thereby compensate for reduced ankle proprioceptive input, so that the effective functional mobility may be achieved.

One novel finding of the current study is that there was a significant correlation between ankle proprioception and step length and step cadence of the 10 WMT. Shortened step length and increased step cadence have been considered the most limiting factors in PD, and important factors that reflected as bradykinesia gait seen in PD (37, 38). The results from the current study suggest that poorer ankle proprioception may be not related to reduced walking speed, but rather, more related to altered gait patterns in PD. In other words, the shuffling gait observed in people with PD may be associated with impaired ankle proprioception. In clinical practice, auditory and visual cues have been recommended as an effective intervention for people with PD to improve their gait (39). Rhythmic auditory cueing is used to improve the temporal parameters of gait, and the spatial parameters of gait are enhanced by accessing visual cues (40). It is possible that auditory and visual cues are beneficial for the sensory integration process in the CNS (41), and thus, external auditory and visual cues may improve the sensory input and compensate for other proprioceptive impairments in people with PD (42, 43). If this is the case, proprioceptive training, such as Tai Chi, may enhance ankle proprioception and consequently improve gait in PD.

However, the association between impaired ankle proprioception and altered gait in PD observed in the current study did not clarify the causal relationship between the two variables. It is possible that impaired ankle proprioception in people with PD may increase difficulty with optimal placing of the feet during walking, so that people with PD gradually develop the strategy of reducing step length and increasing step cadence. It is also possible that, due to fear of falling, people with PD reduce step length, which may subsequently reduce the range of motion at the ankle joint during walking. According to “use-it-or-lose-it” theory, prolonged disuse of full range of movement of the ankle may consequently result in diminished ankle proprioception (44). If the latter explanation is the case, strategies such as using visual and audio cues to improve step length may be also effective at improving ankle proprioception in people with PD. Nevertheless, future studies are warranted to investigate the causal effects between these variables.

In addition, the significant correlation observed between ankle proprioception and the stage of the modified Hoehn and Yahr indicated that the alteration of ankle proprioception may appear at the early stage of PD. Konczak et al. (8) also noted the possibility that the sensibility of proprioception may change very early in the disease process. Thus, ankle proprioception changes could be used as an indicator for disease progression. Further studies are needed to determine when ankle proprioception starts to decline in PD, and whether ankle proprioception could be used as a biomarker to monitor the progression of the disease and effect of intervention on proprioceptive control in PD.

There are some limitations of the present study. Firstly, we did not record some information about PD patients' disease progression, including the duration of the disease, as well as the most effected side. Second, while we tested the patients when they were optimally medicated, we did not record the exact medication they took, and this may limit the implications of the findings in clinical practice. In addition, participants involved in the current study were individuals with mild to moderate PD, who had a relative high level of function. Therefore, it was unclear if the results obtained from this group would be similar to those with severe PD and low functional mobility. Besides, for performing the step count during the 10 MWT, we used a visual observation method, which might be less accurate than automatic step counting devices. Another limitation is that the TUG and 30s-STS have been used as functional mobility measures in the current study. Although these measures are clinically relevant and meaningful, they may not precisely reflect compensations and deviations in functional mobility. Future study is needed to use more objective measures to explore gait deficits in PD. Finally, the AMEDA approach to proprioception testing has the advantage of assessing proprioception in multi-joint movement, with muscular engagement. However, although this enhances the ecological compatibility of the test with functional mobility, it lacks the purity of isolated single joint proprioception testing, and might miss such deficits within this patient population.

## Conclusion

The lack of relationship between ankle proprioceptive acuity and functional mobility in PD suggests that proprioceptive acuity is not the main limiting factor in mobility in the current group of PD patients. Further study is required to establish whether this is compensated by other aspects of sensory input, rather than ankle proprioception, as this would be consistent with sensory reweighting. In addition, poorer ankle proprioceptive acuity was associated with decreased step length and increased step cadence, suggesting that the shuffling gait observed in PD may be related to impaired ankle proprioception, which has important clinical implications for gait retraining in people with PD. Given that ankle proprioception was significantly and negatively correlated with the stage of modified Hoehn and Yahr, it may warrant being used as an objective biomarker to monitor the progression of PD.

## Data Availability Statement

The raw data supporting the conclusions of this article will be made available by the authors, without undue reservation.

## Ethics Statement

The studies involving human participants were reviewed and approved by the Ethics committee at the Shanghai University of Sport (Approval Number: 2016035). The patients/participants provided their written informed consent to participate in this study.

## Author Contributions

YW collected data and drafted the manuscript. JH participated in the study design, data analysis and helped to draft the manuscript. ZW and JZ helped to study design and data collection. JW, EP, GW, and RA made edits and comments to the manuscript. All authors conceived of the study, read and approved the final version of the manuscript, agreed with the order, and presentation of the authors.

## Conflict of Interest

The authors declare that the research was conducted in the absence of any commercial or financial relationships that could be construed as a potential conflict of interest.
